# PREDICTING ACCIDENTS OF OLDER ADULTS LIVING WITH DEMENTIA USING ASSISTIVE TECHNOLOGIES AND MACHINE LEARNING

**DOI:** 10.1093/geroni/igae098.3555

**Published:** 2024-12-31

**Authors:** Kyung Hee Lee, Eunjin Yang, Ji Yeon Lee, Aeyoung Cho

**Affiliations:** Yonsei University, Seoul, Republic of Korea; Gachon University, Incheon, Republic of Korea; Inha University, Incheon, Republic of Korea; Yonsei University, Seoul, Republic of Korea

## Abstract

Although older adults living with dementia prefer to age in their own homes, they face many challenges such as falls, injuries, and night-time disturbances. Predicting and preventing those challenges could help both older adults and their caregivers to maintain aging in place. This study aimed to develop and validate machine learning models in predicting accident subgroups among individuals with dementia using assistive technologies (i.e., in-home motion sensors and actigraphy). Data were collected from 75 older adults living with dementia and their family caregivers in South Korea. Utilizing 966 days of data on sensors, sleep, and survey from dyads, various machine learning models were developed to predict three accident subgroups—physical impact, night-time behaviors/wandering, and risky behaviors. The performances of five machine learning classification models were compared and important features were extracted. Python 3.9 was used for all analyses. The Gradient Boosting Model excelled in predicting physical impact and night-time behaviors, while CatBoost performed best for risky behaviors, achieving F1-scores of 0.50, 0.71, and 0.87, respectively. Important features were different by type of accidents. For physical impact, caregiver’s age, comorbidity severity, and total sleep time were the three top features; for night-time behaviors/wandering, cognitive function and number of medications were the most important features; neuropsychiatric and depressive symptoms were the most important features for risk behaviors. In addition, activity patterns recorded by in-home motion sensors emerged as important features. The findings highlight the potential of these assistive technologies to identify high-risk individuals, provide a safer environment, and support family caregivers.

